# Bayesian Optimization in the Latent Space of a Variational Autoencoder for the Generation of Selective FLT3 Inhibitors

**DOI:** 10.1021/acs.jctc.3c01224

**Published:** 2023-12-19

**Authors:** Raghav Chandra, Robert I. Horne, Michele Vendruscolo

**Affiliations:** Centre for Misfolding Diseases, Yusuf Hamied Department of Chemistry, University of Cambridge, Cambridge CB2 1EW, U.K.

## Abstract

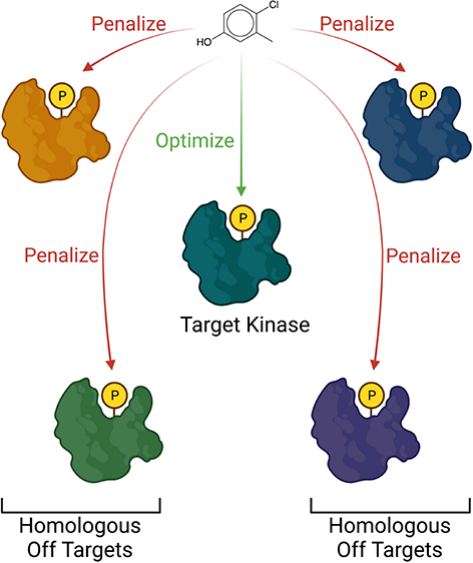

The process of drug design requires the initial identification of compounds that bind their targets with high affinity and selectivity. Advances in generative modeling of small molecules based on deep learning are offering novel opportunities for making this process faster and cheaper. Here, we propose an approach to achieve this goal, where predictions of binding affinity are used in conjunction with the Junction Tree Variational Autoencoder (JTVAE) whose latent space is used to facilitate the efficient exploration of the chemical space using a Bayesian optimization strategy. The exploration identifies small molecules predicted to have both high affinity and high selectivity by using an objective function that optimizes the binding to the target while penalizing the binding to off-targets. The framework is demonstrated for FMS-like tyrosine kinase 3 (FLT3) and shown to predict small molecules with predicted affinity and selectivity comparable to those of clinically approved drugs for this target.

## Introduction

Drug discovery is highly expensive, uncertain, and inefficient.^1,2^ It has been estimated that the development cost of a new drug is close to $2 billion.^1,3,4^ It is also remarkable that 24% of all marketed drugs and 35% of anticancer drugs originated from serendipitous discoveries.^5^ The advent of deep learning methods is providing novel opportunities to address at least some aspects of the issue of reducing the time, cost, and rate of failure of drug discovery pipelines.^6−8^ These approaches frequently require large amounts of relevant data, such as the case of the identification of the experimental antibiotics halicin^9^ and abaucin,^10^ where deep learning methods were trained on the experimentally measured inhibition of thousands of small molecules against bacterial growth.

To reduce the impact of the limitation of the high data requirement, the aim of this work is to develop an end-to-end pipeline for the generation of small molecules with high affinity for a chosen target binding pocket and with low affinity for structurally similar off-target pockets, without the need of target-specific extensive data. The low data requirement of this approach is based on the following observation. A fundamental aspect of generative modeling is to use a deep learning strategy to estimate a function that assigns a probability for a given compound to bind its intended target. Knowing this function enables one, at least in principle, to sample the chemical space to identify compounds with predicted high affinity. However, there are at least two major problems with this approach. The first is that the chemical space relevant for drug discovery has been estimated to contain some 10^60^ compounds,^11^ and thus learning the binding affinity function requires substantial amounts of data. The second is that molecular representations are often discontinuous,^12,13^ and thus not easily amenable to efficient searches.

In order to overcome these problems, one can work in the latent chemical space, which is the vector space used by variational autoencoders in deep-learning methods to represent a compound.^12,13^ Since the size of a typical latent space can be on the order of just 10^2^, one can ask whether learning the function in the latent chemical space could require less data. Furthermore, since in the latent space of variational autoencoders (VAEs), small molecules corresponding to similar vectors are structurally similar, the search for binders of high affinity and specificity can be cast as one of optimization.

**Figure 1:**
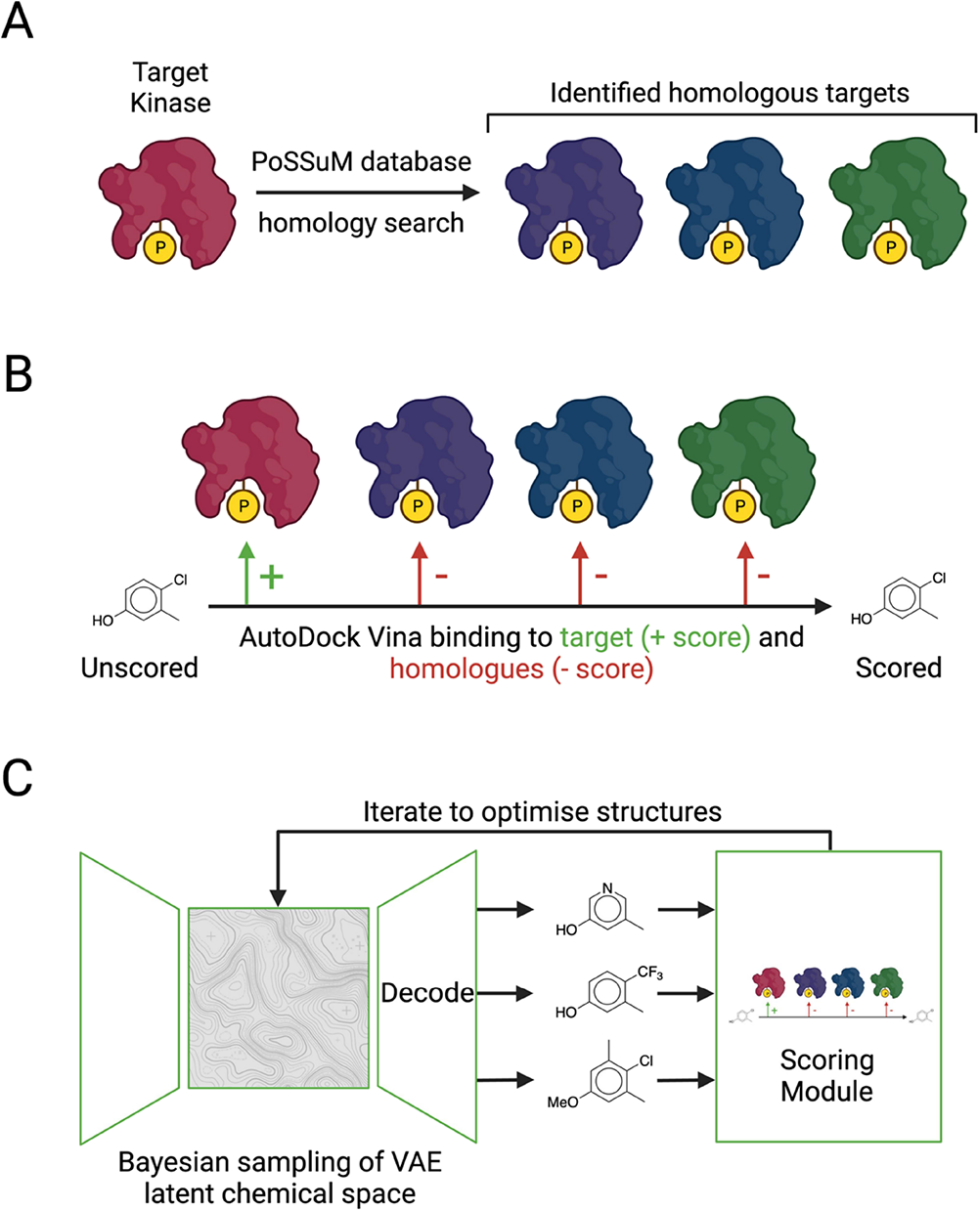
Schematic overview of the method reported in this work. (A) Off-target identification module. After target selection, the PoSSuM^25^ database of similar protein–ligand binding pockets was used to screen for homologous off targets. (B) Scoring module. AutoDock Vina^32^ was applied to screen compounds to predict the binding energies to the binding pocket of the target and to the binding pockets of the homologous off-targets. The binding energies were then used in an objective function that rewarded binding to the target and penalized off-target binding, to provide an overall score for each molecule (see Eq 1). (C) Overall iterative specificity pipeline. Compounds were initially randomly sampled from the latent space of a pretrained junction tree variation autoencoder (JTVAE).^35^ The resulting compounds were then passed through the scoring module, and the latent space of the molecular representations was then iteratively sampled via Bayesian optimization to obtain the molecules that maximized the objective function.

Based on this idea, the pipeline described in this work has 5 components: (1) a method of predicting the binding affinity of a compound for its target pocket, (2) a method of searching for off-target pockets on other proteins similar to the target pocket, (3) a variational autoencoder to represent the structure of the compound, (4) an objective function to estimate the binding affinity and the specificity for the target pocket, and (5) a Bayesian optimization method to maximize the objective function in the latent space of the variational autoencoder. A schematic overview of the pipeline is shown in Figure 1.

This pipeline is illustrated for FMS-like tyrosine kinase 3 (FLT3), which was chosen due to its coverage in the scientific literature, including data on drugs with documented off-target bindings.^14^ FLT3 is mutated in 28% of adult patients with *de novo* acute myeloid leukemia,^15^ and belongs to the class III family of receptor tyrosine kinases (RTKs).^16^ The binding pocket of FLT3 with Gilteritinib bound is what we target here (the ATP binding site). Gilteritinib is, therefore, a type I inhibitor. In this way, we are targeting a known inhibitory mechanism and therefore believe the generated molecules may have relevance as potential type I inhibitors of this particular kinase.

Although several generative methods have been already reported to identify kinase inhibitors,^17−21^ to our knowledge, the problem of specificity for this class of targets^22−24^ has received less attention using generative modeling. Our results indicate that the pipeline that we report is effective in generating small molecules with a predicted high affinity and high specificity for the intended target.

## Results

### Pocket Similarity Search

Naïve optimization of the binding affinity of small molecules against a selected target would confer no selectivity and therefore likely lead to nonspecific small molecules. Therefore, we selected structurally similar protein pockets to be used as off-target test cases. A fast 3D pocket alignment method, PoSSuM,^25^ was used for this purpose (see Methods). PoSSuM uses putative and known binding sites algorithmically determined from the Protein Data Bank (PDB),^26^ and embeds pockets into feature vectors that encode their geometric and physicochemical properties. The cosine similarity of these can then be computed, and 3D alignment is carried out on pairs that have high cosine similarities, indicating that the pockets are similar.

For the test case of FLT3, pockets with cosine similarities greater than 0.8 were sorted by the fraction of identical residues of the aligned residues, and the top five results were selected. The binding pocket used was that of the crystal structure of FLT3 in complex with gilteritinib,^27^ a small molecule binder of FLT3. The top five results after following this procedure were the following 5 tyrosine kinases: PDGFRA (2/18 different residues), CKIT (2/18 different residues), VEGFR2 (5/16 different residues), MK2 (5/16 different residues), and JAK2 (6/18 different residues).

The similarity search was validated using known off-target drugs against FLT3. Several examples of off-target binding are known for the first three pockets above which, like FLT3, are members of RTK class III.^28^ Notably, midostaurin, sorafenib, sunitinib, and other first-generation FLT3 inhibitors bind to these off targets and are not selective for FLT3.^29^ Dual JAK2/FLT3 inhibitors have also been reported.^30^

### Binding Affinity Prediction

The fast and accurate prediction of the binding affinity of a small molecule for a protein is a challenging problem.^1,31^ Here, we adopted a variant of AutoDock Vina,^32^ a widely used docking method to predict the protein–ligand complex structure and the corresponding binding score (see Methods). We found that the variant Vinardo^33^ performs better than the default parameters in all relevant metrics for FLT3. This has a parameter, “exhaustiveness,” which controls how comprehensive the docking procedure is for each molecule. Since the pipeline is modular, other binding affinity predictors could be used.

### Objective Score

We defined an objective score as a function of the predicted binding affinities to the target and off-target (see Eq. 1 and Methods). We did not include any stipulations for drug-likeness in the objective function as it would complicate its optimization, apart from using JTVAE, which was trained on 250,000 druglike molecules from the ZINC database.^34^ The quantitative estimate of drug-likeness (QED)^30^ was calculated for the top-scoring small molecules and found to be high for the majority of them.

### Molecular Representations

We used the latent space of the Junction Tree Variational Autoencoder (JTVAE),^35^ which generates chemically valid small molecules, although not necessarily readily synthesizable or stable (see Methods). The JTVAE encodes and decodes small molecules using a 56-dimensional latent space where each dimension is normally distributed with mean 0 and variance 1. By sampling vectors in this latent space and decoding them, drug-like molecules can be generated.^35^ The published model was used, pretrained on 250,000 drug-like molecules from the ZINC database.^34^

### Bayesian Optimization

Due to the high computational cost of each prediction, which involves a target prediction and five off-target calculations by Vinardo, Bayesian optimization was selected as the method for optimization of the objective function^36^ (see Eq. 1 and Methods). Occasionally (in around 1% of vectors), JTVAE produces small molecules for which RDKit^35,37^ is unable to produce 3D conformations which are required for docking and energy calculations by Vinardo. Empirically, this tends to be due to some chemically unfeasible or synthetically inaccessible substructure, and as such, we are not concerned with missing potential hits due to removing these points. Bayesian optimization is nonstochastic as usually the acquisition function will only have one global optimum, and so an invalid point cannot simply be ignored as optimization of the acquisition function would return the same point. Therefore, target and off-target binding affinities are all arbitrarily set to −5.0 kcal/mol, as this produces a low value of the objective function and discourages exploration of this region. For the case of other pockets, this could be set to the value for ε_anybind_ +1 kcal/mol in the objective function (see Eq. 1 below). The frequency of this event is sufficiently low that it does not significantly impact optimization. In all cases of repeated small molecules, we choose to return the previously calculated scores and binding affinities to prevent the waste of computational resources in repeated re-evaluations.

As the optimization is carried out in a complex and noisy space, the Gaussian process correctly learns that it cannot infer long-range dependencies. In practice, this means that a vast majority of the latent space is stationary. Hence, the typical procedure of optimizing the acquisition function, randomizing a large number of points, and performing derivative-based optimization on them fails to find optima. However, we know that the optima of the acquisition function for such a problem must lie close to the best points that are already known, where the Gaussian process is nonstationary. Therefore, the optimization of the acquisition function is seeded near points where the objective evaluation was greater than 0.4, allowing efficient optimization of the acquisition function. Ten iterations of seeding and optimization of seeded points are carried out using 1024 randomized seeding points and 1024 seeded with a standard deviation of 0.1 in each dimension from points where the objective evaluation was greater than 0.4. These points are optimized using L-BFGS-B^38^ and the point with the highest acquisition value is chosen as the point to sample.

### Targeting FLT3

**Figure 2:**
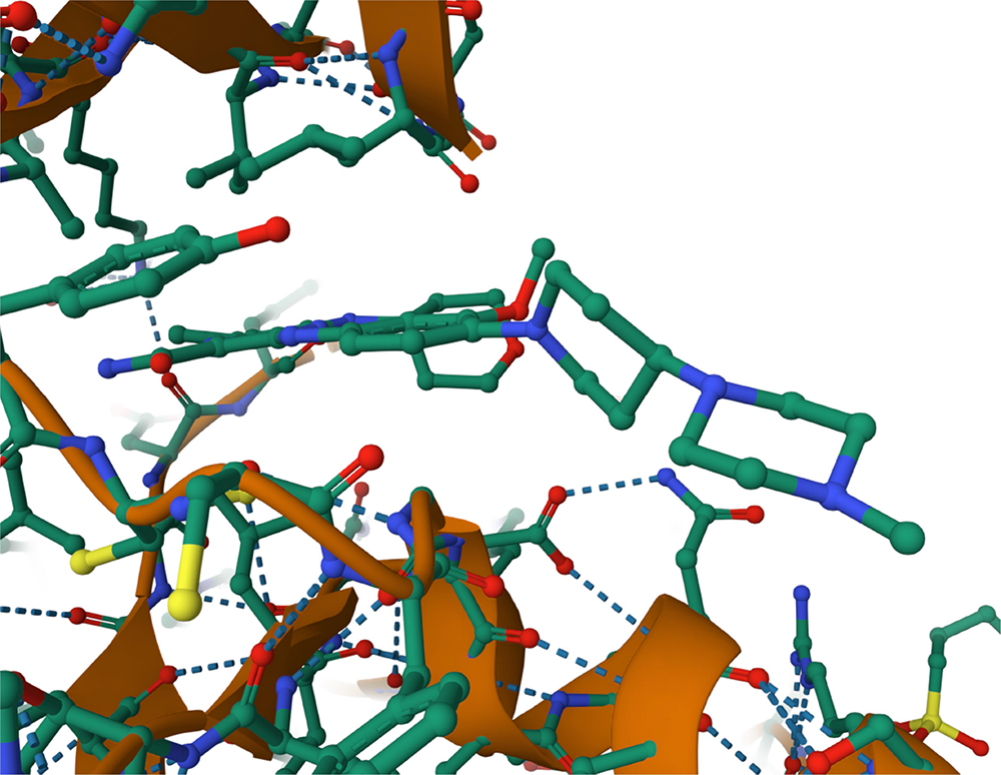
Structure of gilteritinib bound to FLT3. Image from the PDB^26^ of PDB ID 6JQR.^27^

**Figure 3:**
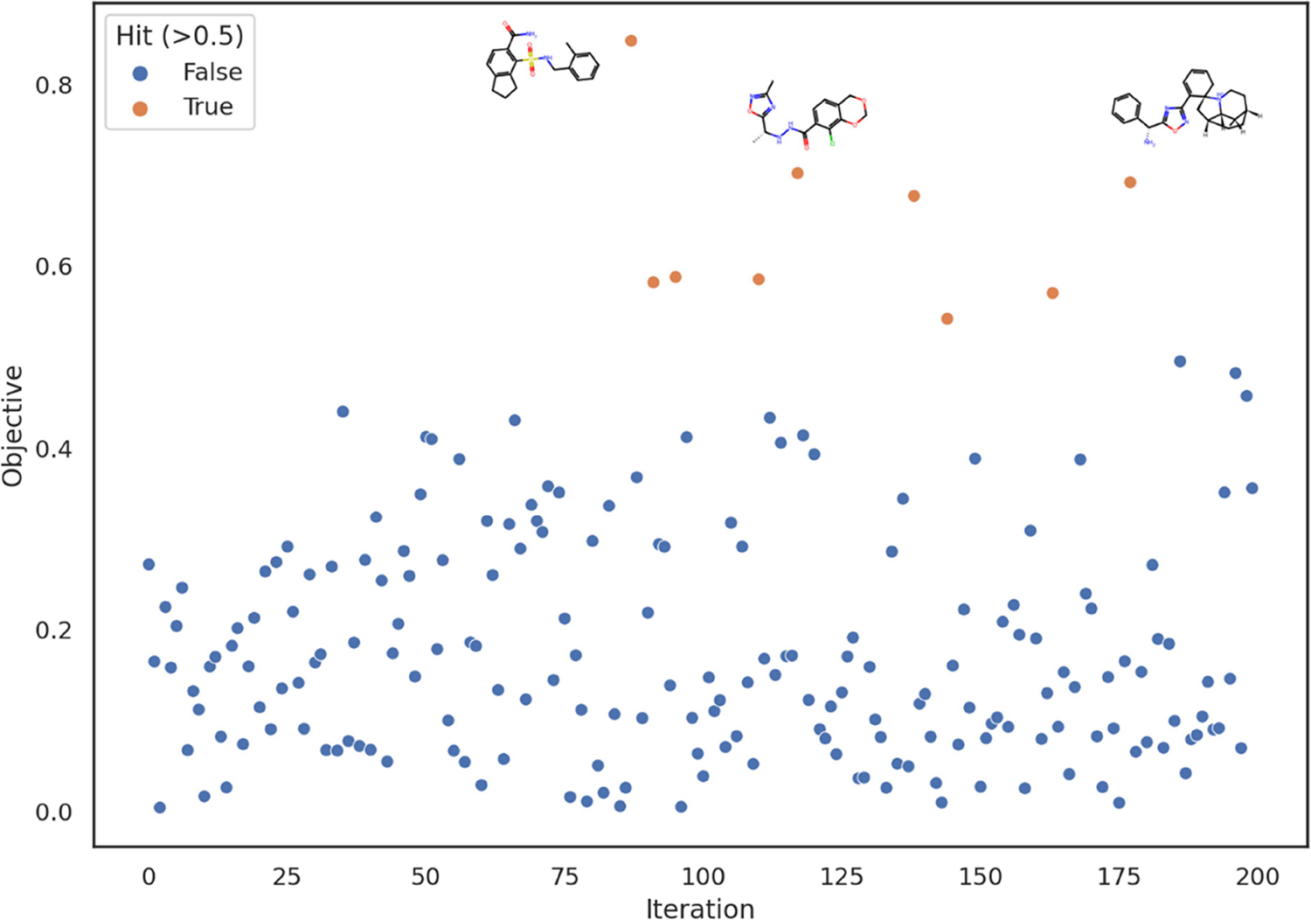
Representative results of a run of 200 iterations of random sampling from the latent space. Hits (shown in orange), which we define to be small molecules with scores greater than 0.5, occur with a frequency of ∼4.5%. Structures of the highest performing 3 small molecules are also shown.

**Figure 4:**
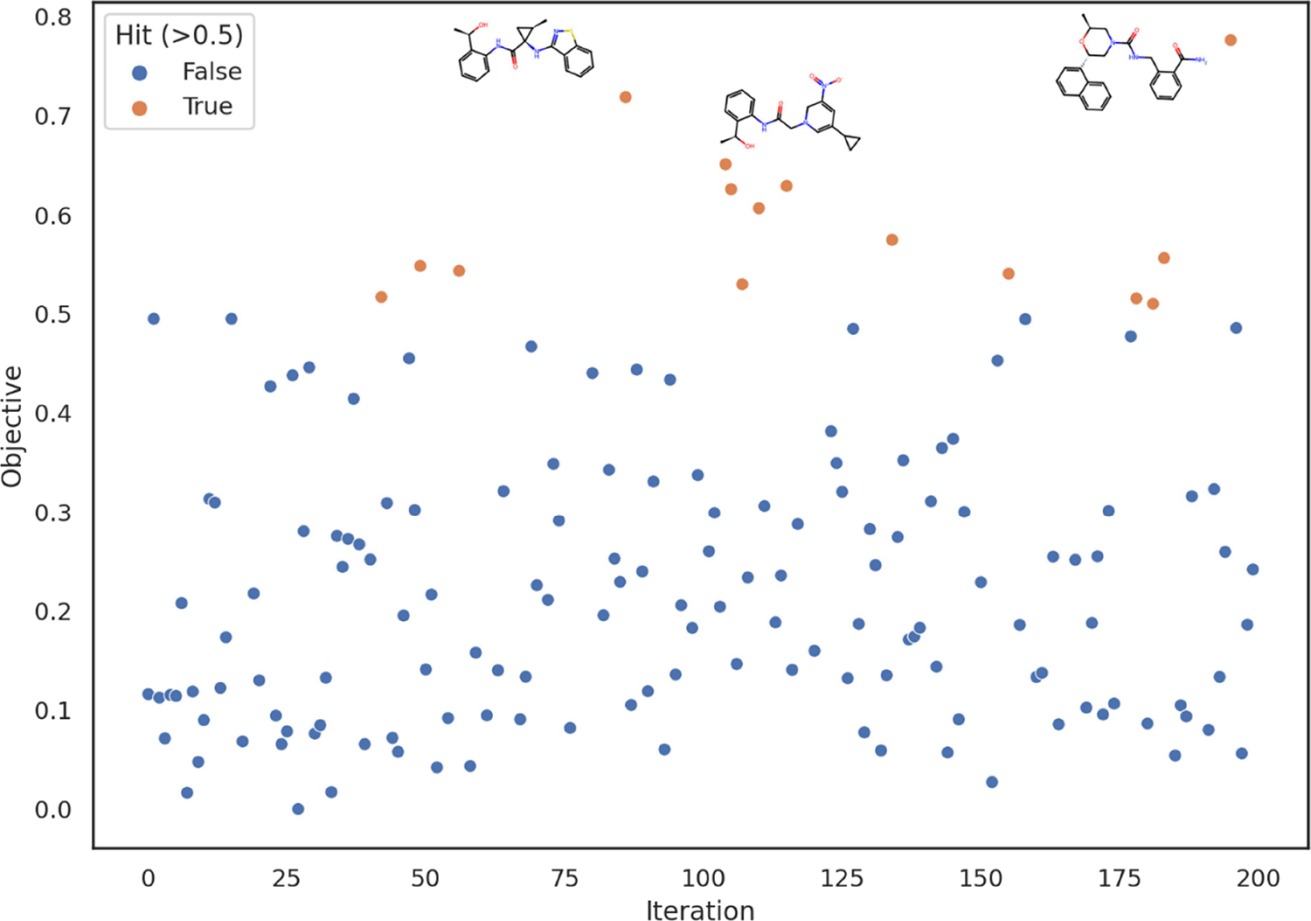
Representative results of a run of 200 iterations of Bayesian optimization following 150 initialization iterations. The expected improvement acquisition function was used with a fixed noise Gaussian process of 0.2 involving the Matern 5/2 covariance kernel. Acquisition function optimization was seeded near points with objective evaluations greater than 0.4. Hits (shown in orange), which we define to be small molecules with scores greater than 0.5, occur with a frequency of ∼10%. The plot displays 156 unique small molecules, and structures of the highest performing 3 small molecules are also shown.

**Figure 5:**
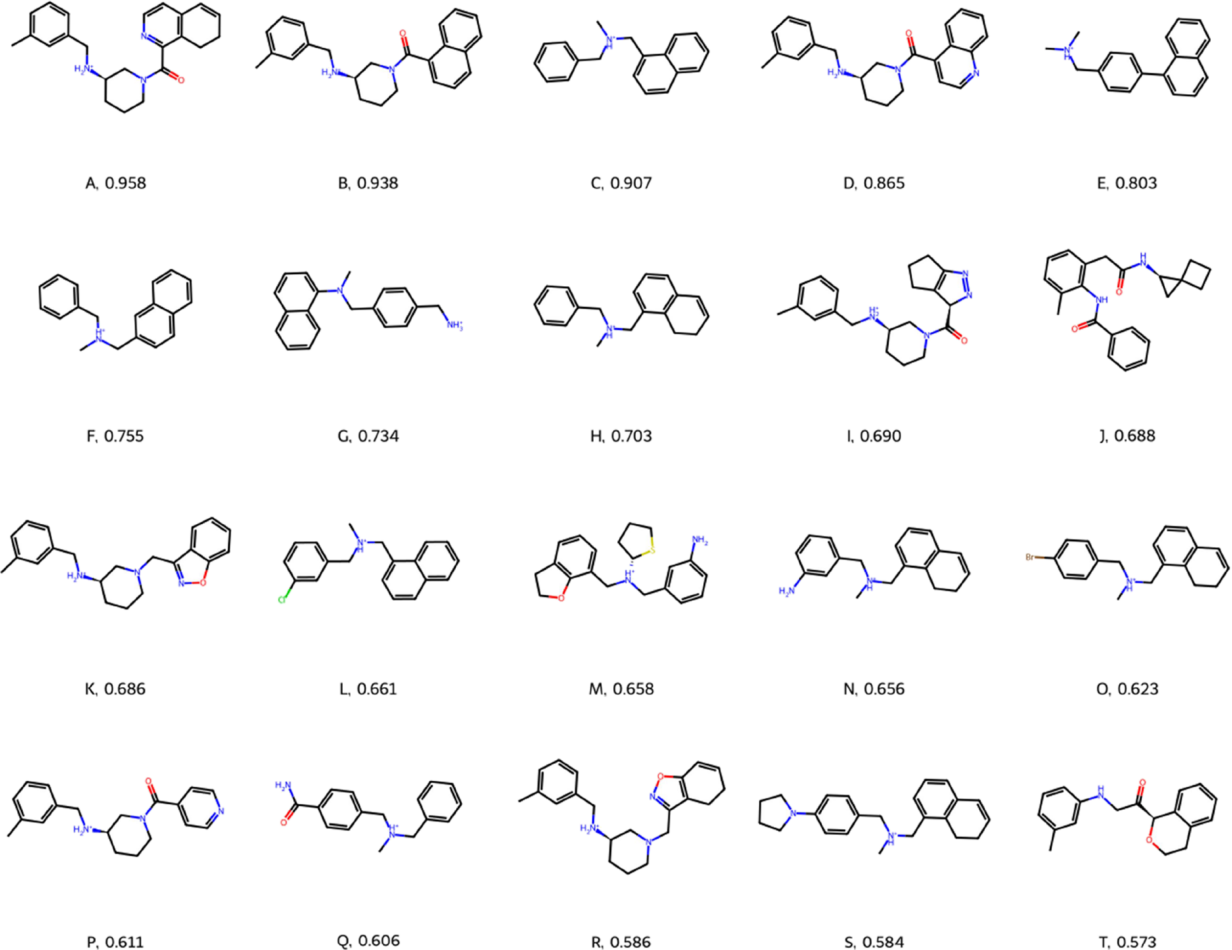
Top 20 predictions after rerunning small molecules with scores greater than 0.5 at 64 exhaustiveness. Small molecules from three runs of 200 iterations of Bayesian Optimisation following 150 initialization iterations.

The structure and binding pocket of FLT3 used are shown in Figure 2, bound to gilteritinib. First, for comparison with Bayesian optimization, random sampling from the latent space was attempted, yielding around 4.5% of samples having objective evaluations greater than 0.5, which was taken as the threshold for being a hit (Figure 3). In all plots, repeated sampling of the same small molecule is not shown. The objective function is noisy and hence a significant fraction of hits from random sampling will be results of fortuitous noise. Bayesian optimization was carried out following a random initialization phase, increasing the hit rate to around 10% (Figure 4). In the first ∼40 iterations, the optimization probes near a variety of points with high objective evaluations, many of which do not give strong evaluations, suggesting that the initial high scores were the results of fortuitous noise. The optimization then converges on a promising region of the latent space for which it generates several high-scoring candidates which are likely not results of fortuitous noise due to their high frequency and structural similarities. Repetition of the optimization with different initializations resulted in the convergence to different regions of the latent space. We thus generated candidates with high levels of structural diversity (Table 1 and Figure 5).

**Table 1:** AutoDock Vina (Vinardo) Binding Affinity Predictions (kcal/mol) at 64 Exhaustiveness for Top 20 Generated Small Molecules across Three Runs with Objective Scores as Defined in Eq 1

**molecule**	**kinase pocket AutoDock Vina binding energies/kcal mol**^**–1**^						**metrics**	
	*FLT3*	*CKIT*	*PDGFRA*	*VEGFR2*	*MK2*	*JAK2*	*score*	*QED*
A	–9.11	–6.02	–6.48	–5.97	–6.14	–5.48	**0.958**	0.909
B	–10.26	–8.44	–6.97	–7.26	–4.62	–5.25	**0.938**	0.760
C	–8.25	–5.27	–4.58	–5.85	–5.60	–6.13	**0.907**	0.737
D	–9.49	–7.93	–6.33	–5.00	–5.89	–7.80	**0.865**	0.778
E	–8.56	–6.23	–5.21	–5.72	–5.99	–7.49	**0.803**	0.739
F	–8.00	–6.45	–4.96	–6.06	–5.27	–6.80	**0.755**	0.737
G	–7.70	–6.41	–5.02	–5.95	–5.21	–6.23	**0.734**	0.780
H	–7.48	–5.39	–4.40	–6.07	–5.55	–6.29	**0.703**	0.866
I	–7.85	–6.87	–4.52	–6.05	–3.9	–6.64	**0.69**	0.899
J	–7.50	–6.26	–4.97	–5.96	–5.79	–5.87	**0.688**	0.862
K	–8.06	–7.19	–5.83	–5.96	–5.98	–6.54	**0.686**	0.779
L	–8.05	–5.96	–7.41	–6.06	–4.31	–6.21	**0.661**	0.749
M	–6.98	–5.46	–4.76	–5.45	–4.62	–5.54	**0.658**	0.822
N	–7.67	–6.68	–4.85	–6.23	–6.27	–5.84	**0.656**	0.827
O	–7.58	–5.12	–6.22	–6.21	–5.85	–6.67	**0.623**	0.865
P	–7.60	–6.88	–5.68	–5.37	–5.38	–6.42	**0.611**	0.935
Q	–6.93	–5.80	–5.21	–5.48	–5.00	–5.26	**0.606**	0.822
R	–7.86	–7.27	–6.00	–5.74	–4.12	–6.86	**0.586**	0.912
S	–7.41	–6.04	–6.14	–6.37	–4.34	–6.36	**0.584**	0.880
T	–7.30	–6.41	–6.06	–5.74	–4.34	–6.20	**0.573**	0.934

### Evaluation of the Generated Small Molecules

Binding affinities of some clinically approved drugs used against FLT3 were predicted at an exhaustiveness of 64 for the purpose of comparison with generated small molecules (Table 2). Only type I inhibitors are shown, as these bind to the same pocket as gilteritinib whose complex structure was used to determine the pocket locations, while type II inhibitors have a different binding site.^39^ The predictions were compared to literature information of known binding affinities to FLT3 and the off-targets^40,41^ (Table 3).

**Table 2:** AutoDock Vina (Vinardo) Binding Affinity Predictions (kcal/mol) at 64 Exhaustiveness for Selected Type I Inhibitors of FLT3 with Objective Scores as Defined in Eq 1

**drug**	**kinase pocket AutoDock Vina binding energies/kcal mol**^**–1**^							**metrics**
	*FLT3*	*CKIT*	*PDGFRA*	*VEGFR2*	*MK2*	*JAK2*	*score*	*QED*
gilteritinib	–5.55	–5.19	–4.73	–4.78	–4.62	–5.91	**0.162**	0.428
crenolanib	–7.33	–6.33	–4.93	–5.36	–4.99	–6.06	**0.657**	0.504
sunitinib	–6.95	–6.79	–5.08	–5.58	–4.92	–6.47	**0.378**	0.626
lestaurtinib	–7.86	–6.19	–5.67	–6.17	–5.59	–7.72	**0.492**	0.373
midostaurin	–7.85	–6.36	–6.06	–6.25	–5.16	–7.05	**0.645**	0.287

**Table 3:** *K*_d_ Values (in nM) for Selected Type I Inhibitors of FLT3 with Scores Estimated Using Eq 3

**drug**	**kinase pocket *K***_**d**_**values/nM**							**metrics**
	*FLT3*	*CKIT*	*PDGFRA*	*VEGFR2*	*MK2*	*JAK2*	*score*	*QED*
gilteritinib	7.0						**1**	0.428
crenolanib	0.74	78	3.2				**0.806**	0.504
sunitinib	0.41	0.37	0.79	1.5			**0.345**	0.626
lestaurtinib	8.5	150	380	220		3.7	**0.293**	0.373
midostaurin	11.0	220	380			94	**0.836**	0.287

We defined an objective score as a function of the predicted binding affinities to the target (ε_target_) and off-targets (ε_off-target_) and an artificial “anybind” target (ε_anybind_). The energy of the anybind target was empirically set to be 1 kcal/mol lower than the average observed binding to the target when randomly sampling from the latent space in order to remove weak binders to the target from consideration. The objective score is defined as
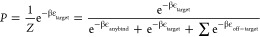
1

**Figure 6:**
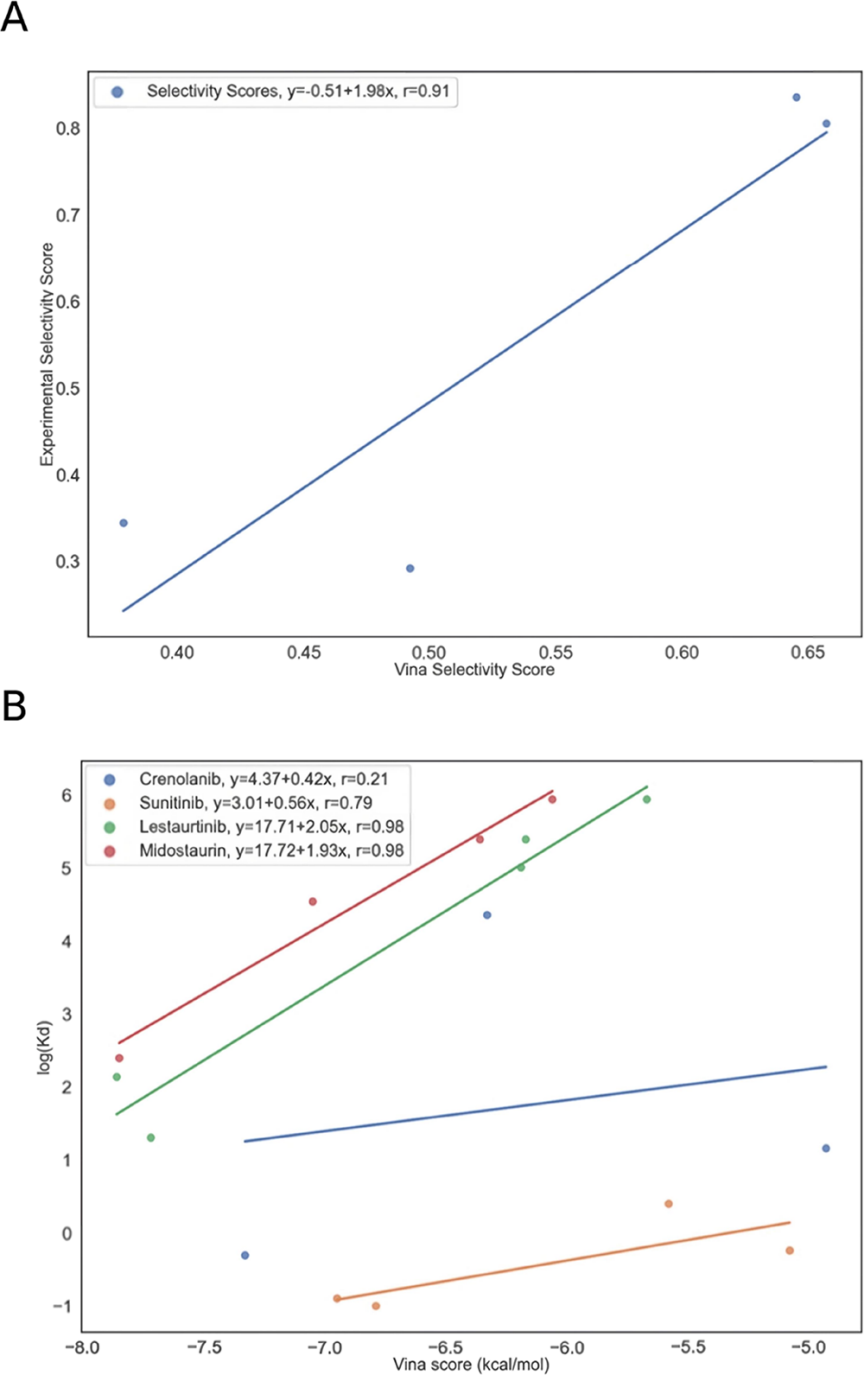
Correlations between predicted and experimental selectivity and binding affinity. (A) Selectivity scores calculated from experimental measurements (Eq. 3) against those from Vina-predicted binding affinities (Eq. 1). (B) Experimentally determined binding affinities against Vina predicted binding affinities.

Using binding affinities and
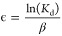
2the objective function (Eq. 1) can be approximated to
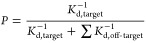
3if it is assumed that the temperature of binding assays was close to 310 K and contributions to *Z* from unreported off-targets and the anybind term are negligible. Carrying out this conversion yields the predictions as indicated in Table 2 and the agreement with those derived from Vinardo is good, aside from gilteritinib (Figure 6A).

Additionally, it was expected that Vinardo binding affinities should be proportional to ln(*K*_d_) and this hypothesis was tested (Figure 6B). The error in the Vinardo predictions is linked to the number of rotatable bonds present in the small molecule^42^ and this is thought to explain its failure for gilteritinib, which has 9 rotatable bonds. The most successful predictions, midostaurin and lestaurtinib, have 1 and 3 rotatable bonds, respectively. The majority of the small molecules generated with this pipeline have fewer than 5 rotatable bonds.

It should be stressed that FTL3 was chosen as a target for its difficult selectivity, as PDGFRA and CKIT both differ by only two out of 18 aligned residues. Therefore, the problem of the distinction between the binding affinities to these pockets is especially difficult. It would be reasonable to expect that a more typical binding pocket could have more dissimilar off-targets, for which this distinction is easier.

Bayesian optimization was carried out three times, each time with 150 random initialization iterations followed by 200 optimization iterations. All hit small molecules (those with objective scores above 0.5) had their objective function rerun with a Vinardo exhaustiveness of 64 and the top 20 small molecules following this procedure are shown in Figure 5 and Table 1. These score comparably or more strongly than clinically approved drugs in all metrics (Table 2). They exhibit high QED scores and selectivity scores as well as strong predicted binding to FLT3. The high QED scores are believed to be inherent to the latent space of the variational autoencoder. Many of the predicted small molecules are amines, which were protonated with reference to a physiological pH.

## Conclusions

We have shown that Bayesian optimization in the latent space of a variational autoencoder is a powerful approach for the generation of small molecules predicted to be highly selective against their chosen target. The generated small molecules for the illustrative case of FLT3 were shown to be comparable, or in some cases superior, in all predicted metrics to clinically approved drugs, including drug-likeness, selectivity, and target binding affinity.

We believe that the pipeline demonstrated here represents a useful method for the generation of hit compounds at low computational expense when compared with strategies such as *in silico* high throughput screening of compound libraries. Importantly, our method encourages selectivity for the desired target, as this aspect is often neglected in computational approaches. We note that experimental validation will be the next step for this work to determine whether the generated small molecules are indeed selective.

## Methods

### Binding Affinity Prediction

We used Vinardo,^33^ a variant of AutoDock Vina,^32^ a common docking method to predict the protein–ligand complex structure and the corresponding binding score. This has a parameter, “exhaustiveness,” which controls how comprehensive the search is. Rigid receptors are used with an exhaustiveness of 8 for optimization which is increased to 64 for subsequent validation of strong candidates.

### Molecular Representations

We used the Junction Tree Variational Autoencoder (JTVAE).^35^ The JTVAE encodes and decodes small molecules using a 56-dimensional latent space where each dimension is normally distributed with mean 0 and variance 1. The published model was used, pretrained on 250,000 drug-like molecules from the ZINC database.^34^

### Pocket Similarity Search

We used the freely available database from a fast 3D pocket alignment method, PoSSuM.^25^ This was constructed using putative and known binding sites algorithmically determined from the Protein Data Bank (PDB),^26^ and embedding pockets into feature vectors that encode their geometric and physicochemical properties. The cosine similarity of these can then be computed and 3D alignment is carried out on pairs that have high cosine similarities, indicating that the pockets are similar.

### Objective Score

We defined an objective score as a function of the predicted binding affinities to the target (ε_target_) and off-targets (ε_off-target_) and an artificial “anybind” target (ε_anybind_), see Eq 1. This score is based on the fraction of small molecules (*P*) that would be bound to the target pocket in the canonical ensemble, where it is assumed that the partition function (*Z*) is comprised of equally weighted Boltzmann terms for the target, off-targets, and an artificial anybind target. The energy of the anybind target was empirically set to be 1 kcal/mol lower than the average observed binding to the target when randomly sampling from the latent space, −6 kcal/mol in the case of FLT3. Any bind target was present to penalize the event of weak binding to the desired target. This can be interpreted as an expectation that any small molecule will be able to find some environment where it will bind with that energy, and it biases the distribution of small molecules so that only those that bind more strongly than this value can have high values of the objective function. For the case of other binding pockets, this constant could either be specified using domain knowledge or set close to the average observed value after random sampling. β is equal to 1/*kT* where *k* is Boltzmann constant and *T* is the absolute temperature, here taken as 310 K. In theory, it would be possible to use protein abundance databases to appropriately weight Boltzmann terms.^43^ However, in practice, abundances differ by several orders of magnitude across databases, so the simpler approach of equal weighting is taken here. Unequal weighting could be used if there were large differences in the magnitudes of target abundances. For the test case of FLT3 and its identified off-targets, this is not the case.

### Bayesian Optimization

Bayesian optimization was carried out using the expected improvement acquisition function^44^ and the ARD Matérn 5/2 kernel^36^ with a fixed noise of 0.2. As we choose to return previously calculated scores for small molecules already seen, an automatic determination of the noise would incorrectly infer very small noise as repeated sampling in the same region would return exactly the same score. Returning calculated scores guides the Gaussian process to learn appropriately large characteristic length scales, which encourage sampling of new small molecules. Optimization is carried out in the unit hypercube, which is cast to the normally distributed space of JTVAE using inverse transform methods.

## Accession Codes

The full code can be found at the GitHub repository: https://github.com/raghavchandra123/selectivebayes.
